# Glacial Southern Ocean deep water Nd isotopic composition dominated by benthic modification

**DOI:** 10.1038/s41598-025-86350-y

**Published:** 2025-01-20

**Authors:** Moritz Hallmaier, Eva M. Rückert, Yugeng Chen, Jasmin M. Link, Riccardo Lizio, Gerrit Lohmann, Marcus Gutjahr, Norbert Frank

**Affiliations:** 1 Institute of Environmental Physics, Heidelberg University, Im Neuenheimer Feld 229, 69120 Heidelberg, Germany; 2 Present Address: GEOMAR Helmholtz Centre for Ocean Research Kiel, Wischhofstraße 1-3, 24148 Kiel, Germany; 3 Present Address: Institute of Oceanography Hamburg, Bundesstraße 53, 20146 Hamburg, Germany; 4 Alfred Wegener Institute, Helmholtz Center for Polar and Marine Research, 27570 Bremerhaven, Germany; 5 University of Bremen, 28359 Bremen, Germany

**Keywords:** Southern Ocean, Ocean circulation, Neodymium isotopes, Benthic flux, Carbon storage

## Abstract

The deep Southern Ocean (SO) circulation plays a key role in the storage and release of CO_2_ in Earth’s climate system. The uptake and release of CO_2_ strongly depend on the redistribution of well and poorly ventilated deep ocean water masses. Recently, evidence was found for possible stronger Pacific deep water overturning and subsequent intrusion into the SO during periods of reduced AMOC. Here, we present new authigenic neodymium isotope data (ɛNd) from two sites within the Atlantic sector of the SO to assess the distribution of water masses during the past 150 ka. PS 1768-8 (3299 m) and ODP 1093 (3624 m) feature unradiogenic interglacial ɛNd-signatures, which are typical for present-day Weddell Sea sourced Antarctic Bottom Water (AABW) (ɛNd ~  − 8.6). During peak glacial periods, radiogenic ɛNd-values ranging from ~  − 2.5 to − 3.5 are recorded. This may be the result of either a strong Pacific or benthic flux influence on the Nd budget in the Atlantic sector of the SO. However, an ocean circulation model indicates no stronger Pacific influence during glacials. Thus, we suggest that an increase in benthic flux influences the SO Nd budget, which is modulated by ACC strength. The more stratified and more sluggish deep water supports decreased vertical mixing and increased glacial carbon storage without the intrusion of poorly ventilated Pacific waters. The occurrence of highly radiogenic glacial bottom water or porewater signatures requires reassessment of the glacial Southern Hemisphere ɛNd-endmember for water mass sourcing reconstructions in the glacial Atlantic.

**Supplementary Information:**

The online version contains supplementary material available at 10.1038/s41598-025-86350-y.

## Introduction

The Southern Ocean (SO) is a crucial component for the uptake and release of atmospheric CO_2_ during glacial-interglacial transitions and recent anthropogenic climate change^1–3^. The increase in respired carbon storage and rapid release through upwelling during deglaciation is presumed to be caused by changes in the presence of differently ventilated water masses in the South Atlantic^1,4–6^. This implies a higher fraction of old, carbon rich water in the realm of presently well ventilated waters, a reduction in deep ocean ventilation via the Atlantic Meridional Overturning Circulation (AMOC), and a reduced CO_2_ flux from the deep SO to the atmosphere during glacial periods^5,7–10^.

Changes in the AMOC through past climate cycles, depth structure, water mass distribution, and leading processes remain debated^4,9–17^. The interaction between the AMOC and SO water masses is even more complex but of major importance, as the Antarctic Circumpolar Current (ACC) connects all ocean basins through the SO^18–21^. NADW flows south in the Atlantic and reaches the ACC at ~ 2–2.5 km water depth^20^. Today, North Atlantic Deep Water (NADW) is situated beneath the Indian Ocean Deep Water (IDW) and Pacific Deep Water (PDW) within the ACC. However, all of these waters are upwelled to the sea surface along bent isopycnals in the SO and diverge to form dense Antarctic Bottom Water (AABW) or Antarctic Intermediate Waters (AAIW) flowing northward^20^.

Through the use of several proxies, evidence was found for possible stronger PDW overturning and an intrusion of Pacific Sourced Water (PSW) into the SO during periods of reduced AMOC^4,17,22–24^. Furthermore, Nd isotope measurements in marine sediments of the SO point to a glacial absence of Weddell Sea Deep Water export into the South Atlantic^10^. Enhanced respired carbon, reduced oxygen and associated increased alkalinity in the deep ocean may thus be related to an increased PSW volume^4^. Nevertheless, the mechanisms driving a PSW invasion into the Atlantic sector of the SO and possibly the South Atlantic currently remain elusive. Moreover, several recent studies in the SO suggested a significant overall slowdown of the ACC during cold and glacial periods^25–27^. Hence, less vigorous Circumpolar Deep Water (CDW) advection in a more stratified SO would also lead to reduced ocean–atmosphere exchange and water mass aging related to an increase of the carbon content in the deep ocean. Due to the relatively rapid onset of strong upwelling and the southward displacement of the ACC during glacial-interglacial transitions, the aged deep water in the SO further bears a high potential to release the respired carbon into the atmosphere.

Neodymium isotopes (given as ɛNd) have proven useful to study deep ocean circulation thanks to basin-scale isotope gradients between the Pacific and North Atlantic^28–31^. PDW is the most radiogenic water mass in neodymium isotopes (ɛNd ~ 0 to − 3) globally^30,32,33^. AABW and AAIW in the South Atlantic (ɛNd =  − 8.6 +  − 0.6^30^) and NADW (ɛNd ~  − 12.5^30^) are far less radiogenic and the SO is a mixture of all three components, represented by (Upper and Lower) CDW (ɛNd ~  − 8.5^34,35^). Nd isotopes stored in the authigenic ferro-manganese coatings of marine sediments may thus provide information toward temporally variable water mass provenances in the Atlantic sector of the SO during past glacial-interglacial climates.

Over the last two decades, the exclusive control of water mass advection on Nd isotopic compositions in authigenic marine sedimentary archives and their applicability for paleoceanographic reconstructions have been debated. Apart from water mass provenance changes, the signature of the authigenic Fe–Mn oxyhydroxide fraction can potentially be altered during early diagenesis associated with benthic isotope exchange reactions after sediment deposition at the seafloor^30,36–39^. Diagenetic processes within the sediment can lead to interactions between detrital and authigenic neodymium (isotopic exchange), and thus to authigenic signatures, which do not represent the water mass isotopic composition. Furthermore, a benthic flux from the sediment may be able to alter the bottom water itself in sluggish circulation regimes, which in turn is reflected in the authigenic phases. This can lead to a water mass Nd isotopic signal, which can be modified by a benthic isotope exchange flux in addition to water mass mixing during advection.

**Fig. 1:**
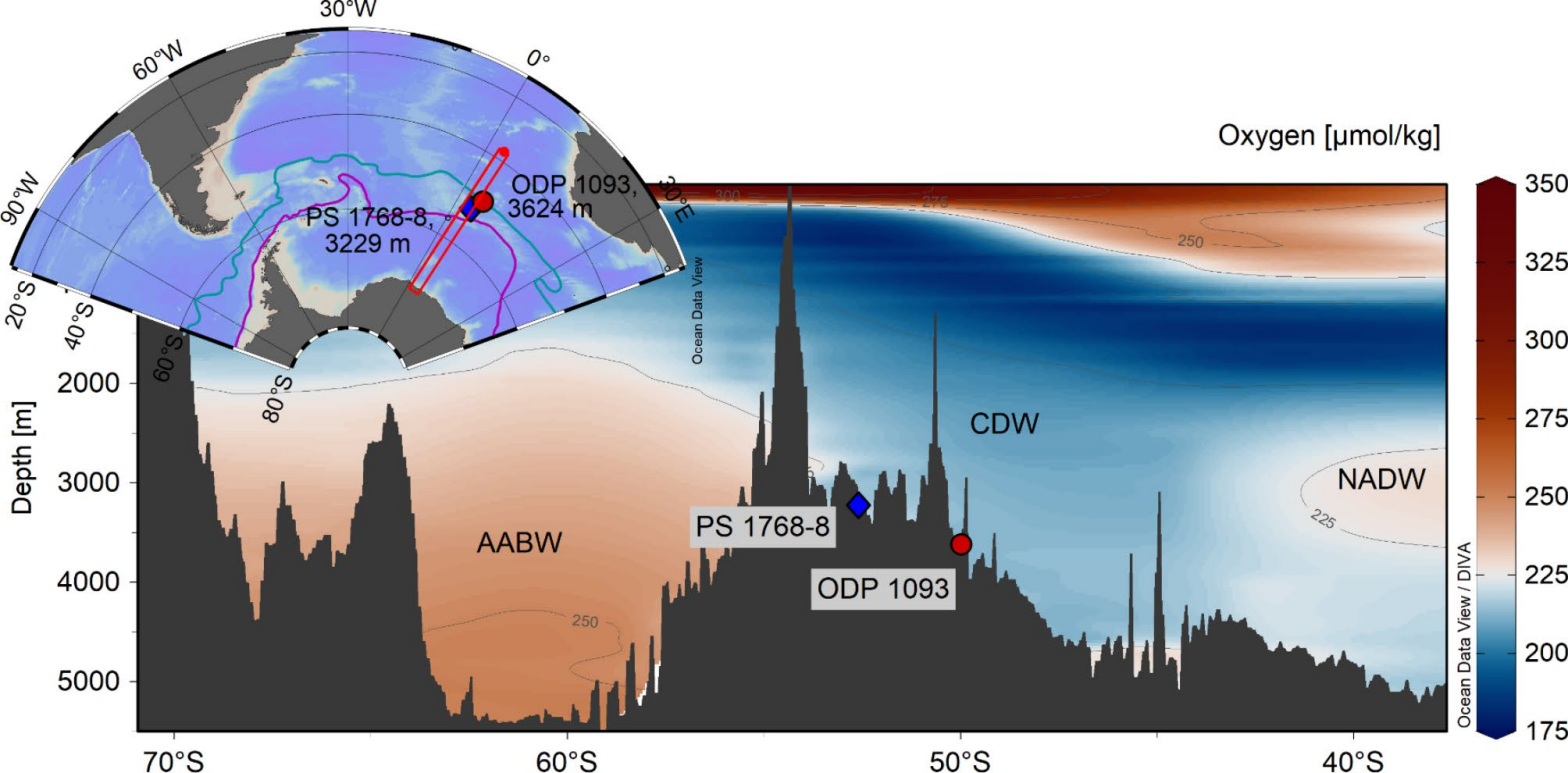
Site locations along a N–S transect at 5°E in the Atlantic section of the SO. Both sites are bathed by LCDW close to the interface of AABW. Both sites are located south of the modern Antarctic polar front. Lines in the map indicate the Antarctic polar front (blue) and the southern boundary of the ACC (purple). Figure created with Ocean Data View^51^. Oxygen data were obtained from the World Ocean Atlas^52^, Bathymetry based on GEBCO 2023 (6 × 6)^53^.

Carbonate sediments have proven particularly useful for reconstructing bottom water ɛNd with low secular influences from non-hydrogenic sources^11,17,40–45^. South of the Antarctic Polar Front pelagic sediment is usually carbonate-free but is primarily composed of diatom ooze, which readily scavenges neodymium on Fe–Mn oxide coatings, whereas the opal itself contains only minor Nd^34,46–49^. Here, we selected ODP Site 1093 (49° 58.60’ S, 5° 51.92’ E, 3624 m) and completed an existing record of PS 1768–8 (52° 35.58’ S, 4° 28.56’ E, 3229 m)^10^, to investigate their Nd isotopic signatures throughout the past 150 ka. Both sites are located south of the modern polar frontal zone within the modern mixing zone of CDW and AABW (see Fig. 1). ODP Site 1093 has high sedimentation rates of 15 to 75 cm/ka, whereas gravity core PS 1768–8 has much lower sedimentation rates of 3 to 30 cm/ka^46,50^. Further details on the age models of both cores are provided in the supplementary material (Fig. S1 and Fig. S2).

**Fig. 2:**
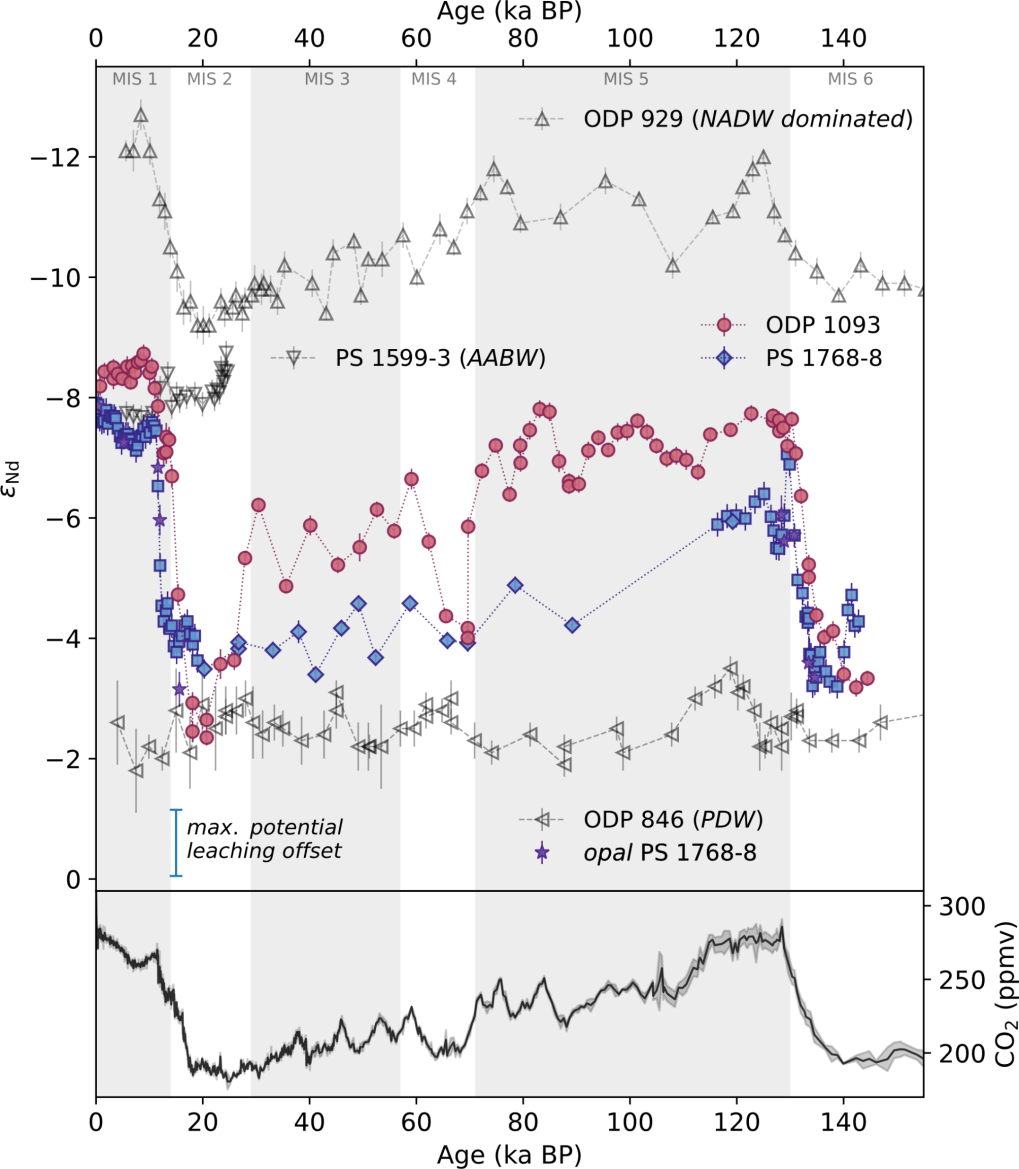
ɛNd records and ice core CO_2_ data from the past 150 ka. Shaded areas indicating marine isotope stages (MIS 1–6)^55^: Upper panel: The records of authigenic ɛNd at SO sites ODP 1093 (3624 m) and PS 1768–8 (3229 m) (also including data from^10^ marked by squares). Furthermore, the equatorial Atlantic (ODP 929)^56^, equatorial Pacific (ODP 846)^32^ and Weddell Sea (PS 1599–3)^10^ ɛNd records are shown in gray. Opal ɛNd from PS 1768–8 is shown as purple stars^10^. The maximum observed leaching offset is indicated as an additional error bar. Lower panel: Atmospheric CO_2_ (shaded area is the 2σ deviation)^57^.

A total of 102 new neodymium isotope measurements provide two continuous 150 ka ɛNd records in the Atlantic section of the SO with intermittent millennial resolution (note that PS 1768–8 is a composite of Huang, et al.^10^ and this study, see supplemental material). These resolve systematic glacial-interglacial variations in the authigenic Nd isotopic composition, with highly radiogenic glacial values that are not reflected in water masses of the modern SO (see Fig. 2).

We further tested the hypothesis that a strong PSW intrusion into the South Atlantic is the main cause of the radiogenic glacial signatures with tracer experiments in the Finite-volumE Sea ice-Ocean Model (FESOM 2.0) (for details see methods). FESOM 2.0 is an ocean-only model, which means that the atmospheric forcing is kept constant. Consequently, this approach has a limitation: it does not account for interactions between the ocean or sea ice and the atmosphere. We utilized the Present-Day (PD) and Last Glacial Maximum (LGM) simulation experiments as described in Chen, et al.^54^. After the simulations reach equilibrium, we introduce tracers into the equatorial East Pacific (seafloor to 1000 m) and continue the simulation for 350 years to observe the distribution changes of the tracer in the Southern Ocean. This strategy allows the quantification of the advection and diffusion of glacial and interglacial PSW into the world’s oceans and thus provides means to detect a potential pure PSW signal in the Atlantic sector of the SO as required to reach the glacial radiogenic Nd values.

### Temporal changes in the ɛNd signatures

**Fig. 3:**
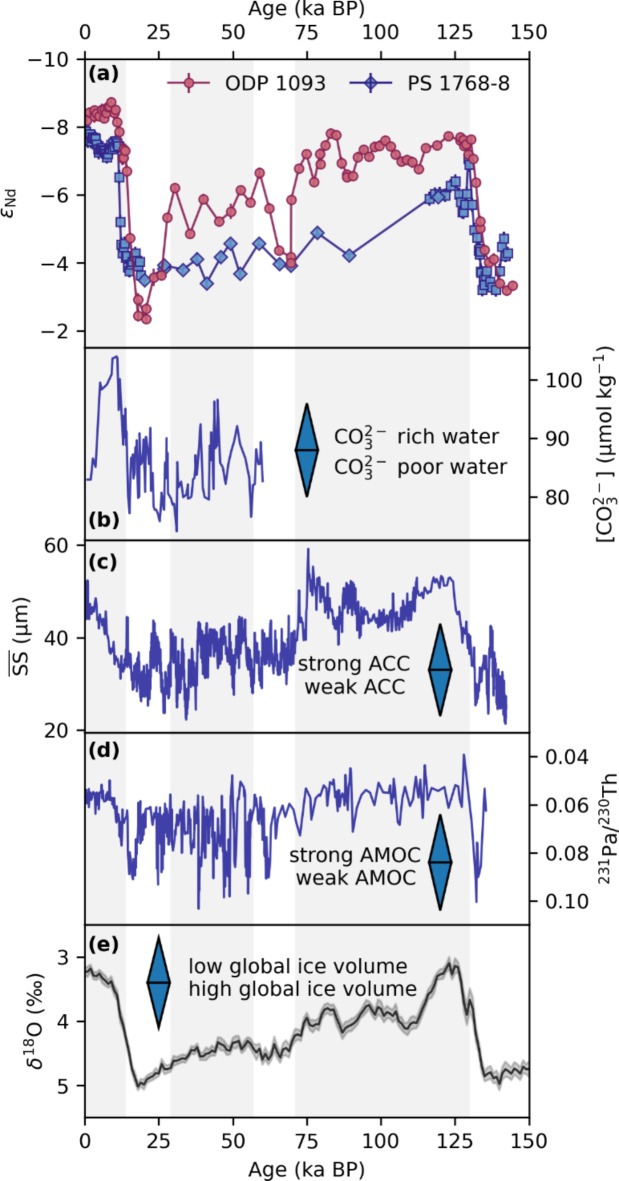
(**a**) SO ɛNd records of site ODP 1093 and PS 1768–8 (the latter is a composite of this study (diamonds) and^10^ (squares), for details also consult Fig. 2) compared with (**b**) the carbonate ion concentration of TN057-21^4^, (**c**) ACC dynamics and flow speed reconstructed based on sediment grain-size in PS 97/085–3^26^, (**d**) AMOC strength at Bermuda rise^11,67–69^, and (**e**) the LR04 δ^18^O as a measure of the global ice volume and deep ocean temperature^55^. This indicates a significant impact of the SO on the carbon cycle during glacial–interglacial cycles.

The two sites examined in the Atlantic sector of the SO indicate substantial temporal changes in ɛNd spanning a range of 6.4 ε-units from − 8.7 during the early Holocene to − 2.3 during the last glacial (Fig. 2). Both yield systematic and synchronous changes in the Nd isotopic composition through the past 150 ka, with slightly less radiogenic ɛNd values in ODP 1093 than in PS 1768–8 during interglacials. The systematic ɛNd patterns match the glacial-interglacial cycles recorded in Vostok ice core and LR04 (Figs. 2, 3). During peak glacial periods of MIS 2, 4 and 6, both sites yielded nearly identical radiogenic ɛNd values ranging from − 2.5 to − 4. During the interglacials of MIS 1 and MIS 5e, both ɛNd records show less radiogenic ɛNd-values of − 6.0 to − 7.8 in PS 1768–8 and − 7.0 to − 8.3 in ODP 1093. At both sites, the compositions observed during MIS 5e are more radiogenic than those observed during the Holocene. The offset between the sites is expanded to almost 2 ε-units during the intermittent warm climate periods of MIS 3 and the end of MIS 5, with less radiogenic values at deeper water depths and/or further north. Both records fall within the bounds of the glacial-interglacial variability of water mass ɛNd values observed between the Atlantic and Pacific Ocean as indicated by the Nd isotopic compositions at the NADW dominated site ODP 929 in the equatorial Atlantic^56^ and at several sites bathed by Equatorial Pacific Deep Water flowing south^32,33^ (Fig. 2).

### ɛNd signatures mainly reflect the extracted authigenic phase

We analyzed the authigenic phase of the sediment, because it is assumed to most likely represent the local bottom water^31,40^. Considering the overall sediment composition, it must be assessed how reliable the sediment phase of interest is extracted during the applied leaching procedure. The leaching of bulk sediments always bears the risk of contamination of the pure authigenic fraction, with soluble Nd from other phases. To minimize such impacts, we used a weak acid leach as proposed by Blaser et al.^40^. In addition, very short 10 s leaching (protocol by Huang, et al.^49^ suggested for low carbonate sediments in the Southern Ocean) revealed significant differences only for samples that were more radiogenic than ɛNd =  − 4 with a maximum offset of 1.1 ɛ-units toward more radiogenic values at site ODP 1093 (see supplement Fig. S3 and indicated in Fig. 2 as an additional error bar). These differences cannot explain the observed climate trends but indicate that the most radiogenic measurements may have been influenced to some small degree by leaching of volcanic material, ice rafted debris (IRD) or lithogenics. Furthermore, overlapping samples from PS 1768–8 with the study of Huang, et al.^10^ show that the leaching protocol used here^40^ did not generate any measurable differences in ɛNd compared with other studies and laboratories. Additionally, we compared our data with foraminiferal data from nearby core TN057-13PC4^58^ and opal data from PS 1768–8^10^ and thus evaluated and confirmed the reliability of the chemical extraction procedure (see supplemental Figure S4). Carbonate, which is most favorable to extract and preserve seawater isotopic compositions^40^, is only abundant in the late Holocene and mid MIS 5 at ODP Site 1093. Nevertheless, carbonate free sections do not coincide with a particular ɛNd-value, as we measured almost the full range of ɛNd values in this study (-7 to − 2) in carbonate free samples (< 3 wt%) (see Fig. S5). IRD and volcanic ash are more abundant in colder climate periods. Volcanic ash at nearby sites TN057-13PC4 and TN057-14PC4 has highly variable and extremely radiogenic (up to ɛNd =  + 8.1) and unradiogenic (down to ɛNd =  − 12.4) signatures^58^ (see Fig. S4). Because the observed authigenic ɛNd records do neither show substantial short-term variability nor such extreme ɛNd signatures as the ash particles, we conclude that the authigenic leachates are only negligibly influenced by direct leaching of volcanic material. The decrease of IRD in PS 1768–8 occurs at a different sediment depth (0.9 m difference) than the rapid change observed in the ɛNd isotopic composition and thus also does not influence the leaching (see supplement, Fig. S6). Dust input (as indicated by Fe flux) is also increased during colder climates^59,60^, as it is also controlled by glacial-interglacial changes. However, almost the whole range of ɛNd values observed at our core sites was measured regardless of whether the sediment sections bear high or low Fe contents (see supplement, Fig. S7 and Fig. S8). Hence, the measured leachates are also independent of changes in the dust content (also see supplement).

We conclude that the leaching of detrital material does not control the observed climate ɛNd variability at our core sites. We were able to exclude the possibility that the authigenic leachates directly reflect changes in the sediment composition and detrital material such as dust, IRD and volcanic glass. In summary, all conducted tests point to a clear authigenic origin of the ɛNd isotopic composition in both studied sediment cores, which does not exclude isotope exchange processes related to potential pore water benthic fluxes.

However, non-hydrogenetic sediment components (e.g., continental detritus, ice-rafted debris, Patagonia-sourced dust or volcanic ash particles) could lead to an alteration of the authigenic coating or even the presumed seawater-derived signal through early diagenetic exchange reactions between the particulate and dissolved phase at the seafloor, known as benthic isotope exchange flux^30,36,37,39,40,61,62^. On the one hand diagenesis can lead to an alteration within the porewater, leading to ɛNd signatures not exclusively representing the bottom water mass, but rather a mixture of accessible detrital phases. On the other hand, a benthic flux from the sediment may be able to alter the bottom water itself, which in turn is reflected in the authigenic coating^36,61,62^. While diagenesis is completely independent of the bottom water signature, a benthic flux can lead to a mixed signal of benthic- and water mass origin^63^. These processes will be discussed in the next section.

### Processes causing the observed authigenic ɛNd signature changes

The authigenic isotopic signatures can be the result of several processes: Either (i) ɛNd represents mainly a water provenance signal, which would imply a major change in ocean circulation with the predominance of a radiogenic water mass of Pacific origin (PSW) at the sites during glacials. Alternatively, (ii) ɛNd signatures may be permanently altered by either diagenesis or a benthic isotope exchange flux, which influences the local bottom water Nd budget imprinting the FeMn coatings. In this case, the observed changes in ɛNd synchronous with climate must represent climatically controlled variations in sediment composition. Finally, (iii) the relative influence of the benthic isotope exchange flux and the provenance signal on the SO bottom water Nd budget changes with time and thus, the ɛNd signatures reflect the alternating dominance between advective water mass influence during interglacials and benthic fluxes during glacials.

The first scenario (i) describes a change in the source of water flowing through the Drake Passage during glacials. It must be noted that the suggested lack of AABW and less southward transport of NADW alone, are insufficient to explain signatures as radiogenic as ~ -3 in the South Atlantic. However, the highly radiogenic ɛNd signatures observed during glacials are still within bounds of modern-day water mass endmember compositions, but nowadays this very radiogenic deep-water mass is present only in the equatorial East Pacific^30,32,33^. Several studies have shown that the NPDW flows southward along the eastern margin of the Pacific Ocean and potentially extends further south along the Patagonian margin into the ACC during glacials^4,32,64^. A stronger Pacific influence during the LGM was also shown in depths of modern LCDW within the Drake Passage by cold water corals^65^. To date, there are no further authigenic ɛNd records along the suggested deep-water flow path, which could record the ɛNd trace of a water mass all the way to the core sites studied here and verify this process. Several sites in the western Pacific^6,66^ measured CDW compositions as ɛNd − 6 ± 1 during MIS 2. However, these sites are too far west and not located within the assumed core of southward flowing PDW but the signature could be explained by recirculated CDW. Additionally, low carbonate ion concentrations were observed during glacials in the deep South Atlantic, interpreted as an expansion of carbon-rich Pacific deep waters into the South Atlantic^4^ (see Fig. 3).

Thus, the observed radiogenic signatures at the sites studied here could be explained by a significant glacial change in ocean circulation with an enhanced deep-water exchange rate of the Pacific and the ACC, along with substantial transfer of highly radiogenic and old PSW to CDW.

**Fig. 4:**
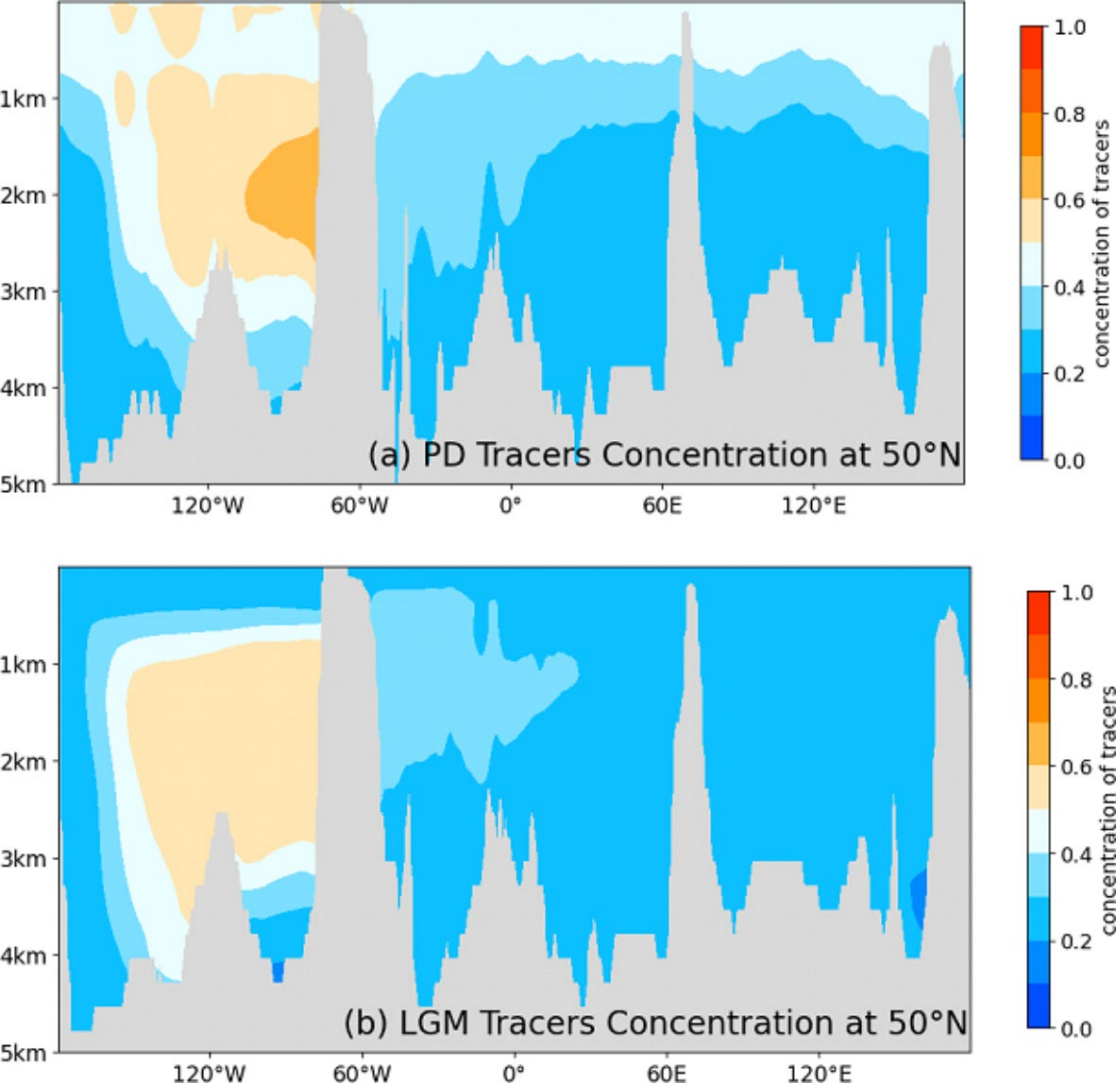
Comparison of dye tracer distributions across a section at 50°S latitude during two different periods: (**a**) The Present Day (PD) and (**b**) the Last Glacial Maximum (LGM). Tracers were added to a stable model and tracked over a span of 350 years to observe their distribution.

However, the interconnection between the Pacific and Atlantic, as well as the ACC flow speed were likely reduced during the last glacial^25,26,70^. The FESOM2.0 simulations reveal minor differences in tracer distribution under glacial and interglacial boundary conditions. Overall, however, the warm climate state provides more efficient transport of the dye tracer into the upper layer ocean, which is expected from the stronger vertical mixing and overturning. Thus, the model rejects that processes, such as sea ice formation in the Southern Hemisphere or changes in the density structure and overturning strength, lead to enhanced transport from the Equatorial Pacific into the Atlantic Southern Ocean and thus, the simulations do not support hypothesis (i) (see Fig. 4 and supplement Fig. S9).

(ii) In the second scenario, the ɛNd signatures are permanently altered by either diagenesis or a benthic isotope exchange flux and the observed variations originate from climatically controlled changes in sediment composition. While a benthic flux has the potential to alter the bottom water mass ɛNd across the sediment-bottom water interface, early diagenesis at the seafloor would influence the preserved Nd isotopic composition independently of the bottom water.

The observed coretop authigenic ɛNd composition at Site 1093 matches the expected present-day seawater composition (-8.6 ± 0.6; see Fig. S4)^30,34^ and is significantly different from the detrital measurement of − 5.2 ± 0.4^71^ (see Fig. S4). This indicates that during the Holocene, the authigenic coating corresponds to the bottom water composition. PS 1768–8 is slightly offset by 0.5 ɛ-units from the seawater value toward more radiogenic values, which might indicate a minor diagenetic influence during the Holocene. During the LGM, both ODP 1093 and PS 1768–8 were close to the detrital LGM value of − 3.8 ± 0.3^71^. Hence, it is likely that diagenetic overprints and a benthic isotope exchange flux played a more significant role during glacials under distinct deep-water circulation regimes.

**Fig. 5:**
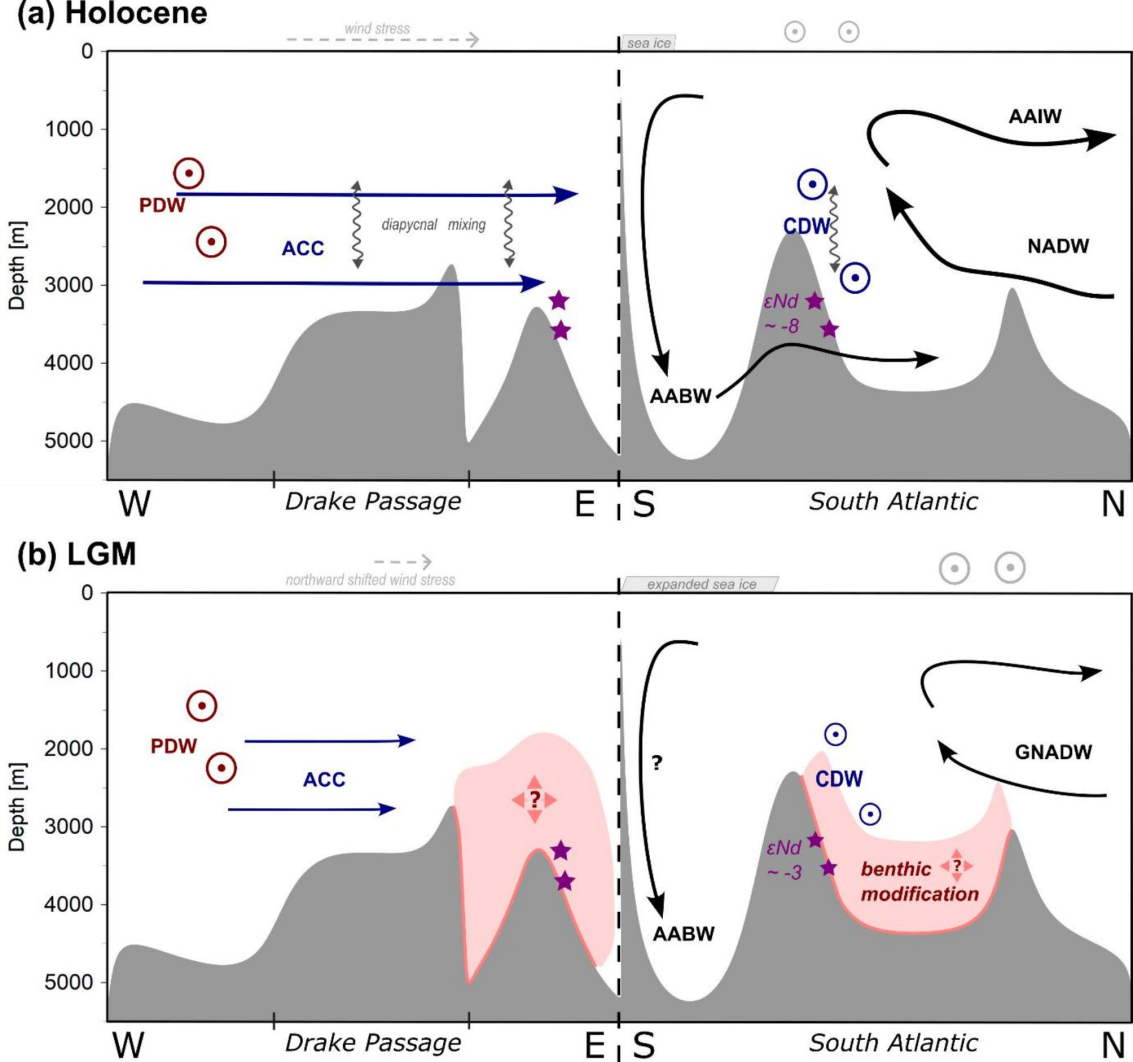
Schematic illustration of the suggested SO circulation regimes and the underlying processes during the Holocene (**a**) and LGM (**b**) along an E–W transect through Drake Passage (sill depth of ~ 2.5 km^70^) and a N–S transect in the Atlantic section of the SO. The distinct radiogenic glacial ɛNd signatures point towards a benthic influence on the Nd budget of sedimentary authigenic signatures and thus most likely the CDW, yet its extent cannot be determined.

Further, a benthic isotope exchange flux has been shown to significantly influence the Nd budget in sluggish circulation regimes^10,38,61,62,72^. The modern SO is dominated by the actively advecting ACC, which forms the most vigorous deep ocean current worldwide. Hence, a large-scale domination of the modern-day bottom-water Nd budget in the SO by a benthic isotope exchange flux seems highly unlikely and contradicts the coretop observation. The renewal time of the water in the SO is on the order of decades, consequently, a massive benthic isotope exchange flux would be required to even alter only the bottommost layer of the water column. Thus, hypothesis (ii) cannot explain the observed variability in ɛNd signatures until there is evidence for a permanent and widespread benthic isotope exchange flux. However, changes in ACC flow speed might regulate the impact of benthic flux in the SO (process iii).

(iii) The impact of the benthic flux could also be modulated by climate dependent parameters. During interglacials, the combined advective ocean dynamics of a strong ACC^25–27^, high AABW production and export^10^ and far southward flowing NADW^16,41,73^ determine the SO Nd budget and the ɛNd signature. However, several studies presented evidence for a glacial slowdown of the ACC^25–27^. Based on the Nd isotopic evolution over the last two terminations, Huang, et al.^10^ suggested that the more sluggish ACC circulation regime during peak glacials allows a more significant control of a benthic isotope exchange flux to the regional Nd budget (see Figs. 3 and 5). Our ɛNd data corroborate this interpretation and further show that ɛNd correlates well with ACC flow speed also for proceeding glacial stages between MIS5 and MIS2 (see Fig. 3). The authigenic ɛNd at Site 1768 seems to be dominated by this benthic weathering reaction throughout almost the entire last glacial cycle. For Site 1093, however, this process seems to be restricted for the interval from MIS2 to the MIS5/4 boundary (Fig. 3). Yet, the volume of the impacted bottom water cannot be determined.

A stronger influence of benthic flux during glacials implies a much less dynamic bottom water circulation regime in the Atlantic Southern Ocean. This is in line with a more stratified SO^21,74,75^ and reduced Weddell Sea bottom water export^10^ leading to the observed aged water mass^4,76^ occupying the deep SO. The model simulation also revealed reduced upwelling of the tracer to the surface water and export to the Atlantic during the LGM (see Fig. 4), which is possibly controlled by augmented sea ice coverage^77^.

To summarize, the observed ɛNd data point to a traceable influence of the ACC flow speed on the bottom water Nd budget during the whole last 150 ka (see Fig. 3). This influence most likely originates from the stronger influence of a benthic isotope exchange flux, which carries radiogenic Nd via pore waters into the bottom water and authigenic FeMn deposits (see Fig. 5). Both studied sites reveal distinct isotopic differences that can be attributed to their relative distances to the southward penetrating NADW and the southern boundary of the ACC. Consequently, ODP 1093 clearly reflects greater influence of unradiogenic Nd from the North Atlantic and is less affected by the radiogenic benthic flux during glacials, with the exception of the extremely radiogenic LGM values. Those are suspected to further reflect diagenesis as an additional source of radiogenic Nd. Overall, we suggest that the global ice volume dependence of the ɛNd signatures reflects the alternating dominance between advective water mass influence and benthic isotope exchange fluxes controlled by the variable strength of the ACC.

### Consequences for the origin of increased carbon storage in the glacial SO

Ice sheets and sea ice were expanded during peak glacials and contributed to a prevention of CO_2_ outgassing and exchange in the SO, northward shifted wind stress and thus reduced upwelling and ACC slowdown^25,75,77,78^. This theory is also supported by ocean circulation models, which demonstrate that the ACC responds sensitively to a reduction or increase in wind stress^78^, similar to locally reconstructed weakened glacial ACC flow speeds applying grain-size measurements (see Fig. 3)^26^. This scenario allows for the presence of a sluggishly moving water mass in the Atlantic sector of the SO storing additional DIC in the deep ocean and explains older ^14^C ages^76^ and low carbonate ion concentrations^4^. Further, the deep ocean is supposed to stratify by salt instead of heat during glacials^12,79^, leading to a decrease in mixing between northern and southern water masses in the Atlantic. The decrease in diapycnal mixing between GNADW and AABW in the glacial Atlantic creates two distinct circulation cells separating the Southern Hemisphere from oceanic heat fluxes more efficiently^12,19^. Further, the salinity of global bottom waters was different during glacials, with the saltiest waters found in the Southern Ocean and a change toward a more saline deep Pacific compared with the deep North Atlantic^12,80,81^.

Overall, we assume that the processes leading to a carbon rich deep water in the Atlantic section of the Southern Ocean during glacials are controlled by a latitudinal shift in the Southern Hemisphere wind-field^78^, a weakening of the ACC^25,26^, a less southward-reaching and/or shallower position of glacial northern-sourced water (NSW)^15^, a more stratified SO^21,74^, a changed glacial density structure^80^, and a reduced or halted AABW export outside of the Weddell Sea^10^. In addition, the coupling of Northern Hemisphere deep water formation and SH winds leads to an adjustment of the geostrophic balance upon reduced NADW production^82^.

Our observations clearly support these leading hypotheses that old and poorly ventilated water masses fill the glacial Atlantic section of the Southern Ocean between depths of 2.8 and 3.6 km: A dramatic shift from advection-dominated authigenic ɛNd isotope signatures with latitudinal differences between northern and southern sites to increasing benthic isotope exchange influences during maximum global ice extent confirms the reduction in flow through the ACC. The exceptionally radiogenic signatures infer a strong benthic isotope exchange flux, potentially with the absence of AABW, and respired carbon increases during periods of global ice volume maximum during the last 150 ka. These periods are synchronous with distinct minima of atmospheric CO_2_ and thus with maximal glacial carbon storage in the SO^4,5,10,22,24^. The higher carbon content in the deep ocean and the associated changes in the pH could additionally favor stronger benthic weathering.

### Rapid carbon release during terminations supported by reestablished iso- and diapycnal mixing

We further observe an abrupt decrease in ɛNd and thus a change from a benthic flux dominated to an advection dominated water mass during terminations (Fig. 2), whereas the glaciations are characterized by a more gradual rise in ɛNd and thus establishment of benthic flux dominated conditions. This process may be accompanied by more variable fractions of advection and benthic flux inputs in the Nd budget at the studied sites, marked by increased ɛNd variability and substantial ɛNd differences (2–3 ε-units) between ODP 1093 and PS 1768–8, as observed during MIS 3 and late MIS 5. Thus, PS 1768–8 may have been dominated by benthic flux during these times, whereas site ODP 1093 was influenced only during peak glacial conditions. 

The relatively abrupt transitions to less radiogenic ɛNd during terminations are not synchronous at PS 1768–8 (12.5–11 ka) and ODP Site 1093 (17.5–14 ka) (see Figs. 2 and S10). This can either be related to age model uncertainties (see supplement) or show a different timing due to the slight differences in water depth and/or latitude. Huang, et al.^10^ further investigated the authigenic Pb isotopic composition of nearby site ODP 1094 and suggested a two-step process, with first a successive southward displacement of the SO overturning circulation cell (captured by authigenic Pb at site ODP 1094 at ~ 17 ka) and second the onset of Weddell Sea Deep Water export (captured in PS1768-8 ɛNd at ~ 12.5 ka) (see Fig. S10). In detail, the shift in ɛNd in ODP 1093 also proceeds in two steps, with the initial shift synchronous with the Pb isotope shift at ODP Site 1094 and the second synchronous with the start of the shift in ɛNd in PS 1768–8 (see Fig. S10). Thus, if the different timing in ɛNd is not related to age model uncertainties, it is likely that, along with the southward displacement of the SO upwelling zone, iso- and diapycnal mixing reestablished relatively early in the more northern latitudes of Site ODP 1093. The onset of vertical mixing and advection of the previously well stratified SO would also lead to efficient dilution of the Nd of the benthic flux dominated water mass and the successive reestablishment of advection dominated ɛNd signatures from the north. The reinvigoration of abyssal water exchange with the overlying water masses would allow carbon-rich deep water to supply wind-driven upwelling in the South Atlantic. The resulting northward transport of the glacial AAIW at shallow to mid depths would lead to the efficient release of CO_2_ to the atmosphere (Figs. 2, 3). The second small shift in ɛNd at ~ 12.5 ka BP is synchronous with the change in ɛNd at PS1768-8 and might be related to the arriving Weddell Sea derived AABW. WSDW today has an ɛNd of − 9 as it leaves the Weddell Sea to the north, whereas LCDW in the Drake Passage has a signature of − 8.2^34^. However, the earlier ɛNd shift in ODP 1093 could also indicate that AABW export started earlier than previously assumed, but was initially limited to deeper water depths and thus not noticed at the shallower core site PS1768-8.

### Benthic flux domination in 3.3 to 3.6 km water depth

**Fig. 6:**
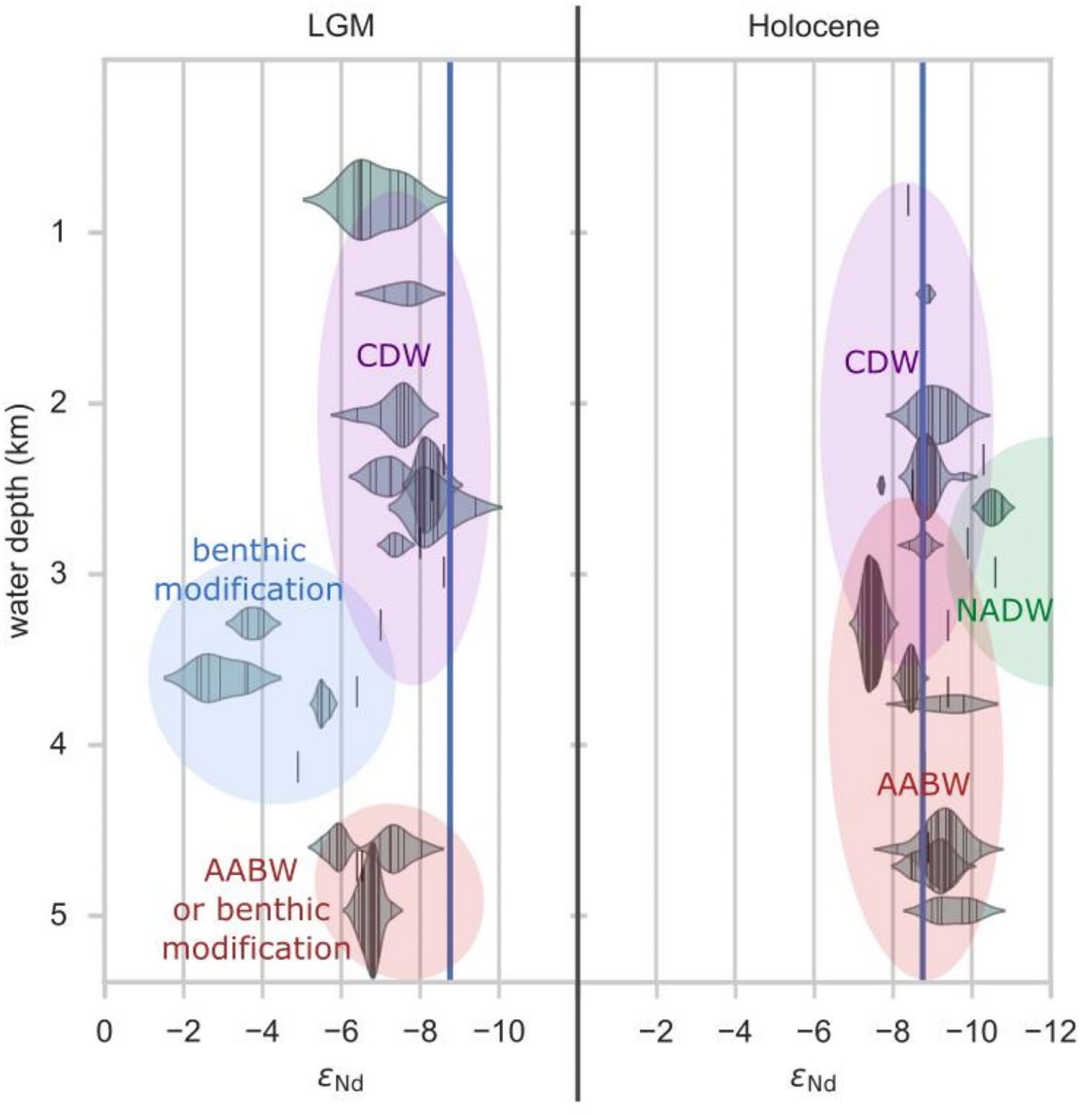
Comparison of the vertical glacial–interglacial ɛNd distribution at different sites in the South Atlantic and Atlantic section of the SO. In 3 to 4 km water depth, there is a strong shift to more radiogenic values at several sites, underlining our hypothesis of a traceable influence of a benthic isotope exchange flux on the sediment–water interface and potentially deep water in the eastern Atlantic SO. The blue vertical line represents the interglacial mean of the collected authigenic ɛNd data (ɛNd =  − 8.76). The distribution of several measurements of one core within the Holocene or LGM period is illustrated by violin plots representing the probability density of the measurements. Less than two measurements per core and time period are illustrated by single vertical lines without violins because of the reduced reliability. The influences of various locations must be considered, as latitudinal and longitudinal differences lead to variations in the ɛNd signal. Detailed information on the compilation can be found in the supplementary material and Fig. S11.

To confine the depth of the benthic influence more precisely, we compared our data to other ɛNd studies of the South Atlantic and the Atlantic section of the SO, however ɛNd data south of the polar front are sparse (see Figs. 6, S11 and dataset). The interglacial period reflects a nearly homogeneous depth distribution with a mean ɛNd of − 8.8 across the entire water column from depths of 1 to 4.5 km. This confirms efficient vertical homogenization in the modern SO and advection dominated ɛNd signatures, as they are in line with direct modern ocean water mass ɛNd^30,34^. During the last glaciation, there was an increase of up to + 5 ɛNd for authigenic signatures at depths below 3000 m, slightly decreasing toward deeper depths below 3900 m. This underlines our hypothesis of a traceable influence of a benthic isotope exchange flux on the sediment–water interface and potentially deep water in the eastern Atlantic SO. Moreover, the more radiogenic ɛNd signatures observed at several sites favor the explanation of a benthic flux dominated regime in the glacial SO over a local diagenetic feature at the here studied sites. Furthermore, if not only the bottom layer of the water column is diagenetically altered, but also the deep water, our results would advocate for a glacial Atlantic Southern Sourced Water ɛNd endmember of ɛNd ~  − 3.5.

At water depths below 4.2 km, ɛNd is less radiogenic (~ -7), which on the one hand could indicate that AABW export at deeper depths is still sufficient to diminish benthic influences and that water mass advection still dominates the Nd budget. On the other hand, the deeper sites of this compilation are located slightly further north, where the detrital material is less radiogenic^28^. Thus, a benthic modification under a sluggish glacial water mass would lead to less radiogenic authigenic ɛNd at these sites. Furthermore, above 3000 m we observe ɛNd signatures of ~ -7, representing glacial CDW, which is slightly more radiogenic than its interglacial counterpart, likely due to a reduced northern influence and/or a slightly more radiogenic GNADW endmember signature advected to the South Atlantic^14^. Thus, we conclude that substantially reduced benthic flux modification is observed at these depths. The mechanisms leading to the observed depth dependency of the benthic flux modification within the Atlantic Southern Ocean require further investigation.

### MIS5e conditions differing from the holocene

In addition to the generally more unradiogenic ɛNd during peak interglacials, both ODP 1093 and PS 1768–8 also reveal differences between the Holocene (MIS 1) and Eemian (MIS 5e) interglacials, with consistently more radiogenic ɛNd (~ + 1 ε-unit) during the warmer MIS 5e (see Fig. 2). Further, the greater difference in ɛNd between ODP 1093 and PS 1768–8 during MIS 5e (~ 1.5 ε-units) than during MIS 1 (< 1 ε-unit) indicates a greater ɛNd range within the SO regime during the penultimate interglacial. The Eemian interglacial MIS 5e approximately 127 ka ago was characterized by sea levels 1–9 m higher and sea surface temperatures 1–2 °C higher than today, reflecting similarities to recent climate change^83^. The ACC speed was likely slightly higher during MIS5e than during the Holocene^25–27^. Thus, it is unlikely that the difference in ɛNd between MIS 1 and MIS 5e is determined by higher benthic influences modulated by ACC flow speed. However, coupled climate models show a great diversity in the ACC response to climate warming and a reduction or latitudinal shift in ACC transport is also reasonable^84^. Further, observations and climate models predict a freshening of surface waters due to increased meltwater input in the SO under anthropogenic climate change, leading to a slowdown or even a shutdown of AABW formation^85–88^. A combination of this circulation slowdown in the Atlantic SO, along with potential additional benthic modification and/or a slightly greater PSW contribution, may lead to the observed modification by 1–2 ɛ-units compared with the present day. In contrast to glacial conditions, a more sluggish flow or a higher fraction of PSW might not necessarily imply increased carbon storage. Under a warmer climate, the deep ocean is still stratified by heat. Since thermal stratification is generally weaker than saline stratification, vertical mixing still occurs, and thus, the water can feed the upper AMOC branch. Additionally, compared with glacial periods, reduced sea ice extents would allow ocean–atmosphere exchange over a larger area. All of these factors may lead to more efficient carbon release into the atmosphere through the SO. However, this is not traced by generally higher CO_2_ levels during the Eemian in ice core records, but the maximum pCO_2_ is reached faster and maintained longer than during the LGM-Holocene transition (see Fig. 2).

## Conclusion

To conclude, we studied two SO sites with oscillating climatically controlled authigenic ɛNd patterns during the last 150 ka, covering two glacial terminations. We tested the hypothesis that the observed strong Nd isotopic shifts are the result of water mass changes, which would suggest the recurrence and persistence of a Pacific-sourced water mass in the South Atlantic during peak glacial climate. Even if this mechanism could explain the observed signatures, the results of two general ocean circulation model simulations under glacial and interglacial forcing revealed no increased PSW transport during the LGM. Thus, the Nd isotopic compositions at abyssal depths in the Atlantic sector of the SO are most likely the result of a stronger influence of benthic isotope exchange flux on the regional bottom water mass Nd isotopic composition in the Atlantic section of the SO, modulated by ACC current strength.

If benthic modification affects not only the sediment–water interface but also the deep water, our results advocate for a glacial Atlantic Southern Sourced Water ɛNd endmember of ɛNd ~  − 3.5. In future studies, it will be important to identify the vertical and regional boundaries of this benthic weathering regime in the glacial Southern Ocean and the hydrodynamic and climatic thresholds above which those are no longer relevant for preserved authigenic Nd isotopic signatures. Furthermore, our results support the presence of a low-oxygen, high-nutrient water mass, which had reduced atmospheric exchange during times of maximum global ice volume. That goes along with an increased storage of respired carbon in the deep glacial South Atlantic and a significant contribution to the increase of atmospheric CO_2_ during terminations. MIS5e shows different ɛNd signatures than those of the Holocene, suggesting visible changes in the SO circulation regime during warmer than present-day climate.

## Methods

### Nd isotope measurements

The applied methodology of extracting the seawater Nd isotope composition from authigenic ferro-manganese deposits is well established and has been carried out with weak acid leaching according to the protocol of Blaser, et al.^40^. Mass spectrometric Nd isotope measurements were performed at the Institute of Environmental Physics multicollector ICPMS facility in Heidelberg, which hosts a ThermoFisher Neptune Plus MC-ICPMS coupled with an APEX HF desolvating system and an ESI-SC autosampler.

To correct for instrumental mass fractionation, the raw ^143^Nd/^144^Nd ratios were double-corrected to ^146^Nd/^144^Nd = 0.7219 and to ^142^Nd/^144^Nd = 1.141876^89^. Further, the influence of ^142^Ce on ^142^Nd and Ce-hydrides interfering with ^143^Nd was corrected with a linear correction depending on the Ce content of the samples, based on measured εNd values of Ce spiked standards with various Ce concentrations. The corrected data were normalized to the JNdi-1 bracketing standard (^143^Nd/^144^Nd = 0.512115^90^). All Nd isotope data are expressed in ɛNd = ((^143^Nd/^144^Nd_sample_)/(^143^Nd/^144^Nd_CHUR_) − 1) × 10 000 with ^143^Nd/^144^Nd_CHUR_ = 0.512638.

The internal errors are the standard error (2SEM) of each measurement consisting of 60 cycles (shown error bars in the figures). The reproducibility of the measurement was ensured by three total procedural replicates of reference material Nod-P-1, yielding a mean value of εNd =  − 3.70 ± 0.31 (2SD), which is in line with published values. The measurement reproducibility between the different measurement sessions was further verified by several total procedural sample replicates (see Dataset 1). Additionally, two in-house secondary standards were measured at a Nd concentration of 50 ppb a total of 72 times, leading to a deviation of the respective mean εNd values of 0.26. Furthermore, the consistency with existing measurements in PS 1768–8 by Huang, et al.^10^ is proven by overlapping samples. Total procedural blank contributions to the Nd concentration were below 0.5% and thus negligible.

### Model simulation

The Finite-volumE Sea ice-Ocean Model^91^, which is the ocean component of the AWI Earth System Model^92^, is employed in our experiments. FESOM 2.0 solves the primitive equations in the Boussinesq and hydrostatic approximations. It adopts an unstructured triangular mesh framework, with scalar degrees of freedom located at vertices and horizontal velocities at triangle centers. Additionally, the Finite Element Sea Ice Model^93^ is incorporated into FESOM 2.0 as a set of subroutines. FESIM solves the modified elastic-viscous-plastic (mEVP) dynamical equations, enabling a reduction in subcycling steps while maintaining numerical stability^94,95^.

For PD simulations, we apply five cycles, each forced by the 1958–2020 period in the Reanalysis dataset (JRA55-do 1.4.0) by Kobayashi, et al.^96^. We detect no significant trend and take the average results from the final cycle for our analysis. The simulations conducted for the PD scenario using FESOM 2.0 have been thoroughly validated. Detailed assessments and descriptions of the PD case’s configuration can be found in Scholz, et al.^97^ and Scholz, et al.^98^. Regarding the LGM simulations, the differences in model configuration are attributed to surface forcing and initial conditions. Both of these two are taken from the LGMW case in Zhang et al.^99^. The LGM simulations are executed over a duration of 600 years to achieve a quasi-equilibrium state.

## Electronic supplementary material

Below is the link to the electronic supplementary material.

Supplementary Material 1

Supplementary Material 2

## Data Availability

Data is provided within the manuscript and supplementary information and dataset. Additionally, it is stored in PANGEA database: 10.1594/PANGAEA.956277.
