# Supplementary material for: Glacial Southern Ocean deep water Nd isotopic composition dominated by benthic modification
Source: Sci Rep. 2025 Jan 20;15:2586. doi: 10.1038/s41598-025-86350-y (PMC11747105; doi:10.1038/s41598-025-86350-y)
Supplement: Supplementary file 2 — Supplementary Material 2 [file 41598_2025_86350_MOESM2_ESM.pdf]

Supplementary Information for

Glacial Southern Ocean Deep Water Nd Isotopic Composition Dominated by Benthic Modification

Moritz Hallmaier[1,2]*, Eva M. Rückert[1,3]*, Yugeng Chen[4], Jasmin M. Link[1], Riccardo Lizio[1], Gerrit Lohmann[4,5], Marcus Gutjahr[2], and Norbert Frank[1]

[1] Institute of Environmental Physics, Heidelberg University, Im Neuenheimer Feld 229, 69120 Heidelberg, Germany

[2] GEOMAR Helmholtz Centre for Ocean Research Kiel, Wischhofstraße 1-3, 24148 Kiel, Germany

[3] Institute of Oceanography Hamburg, Bundesstraße 53, 20146 Hamburg

[4] Alfred Wegener Institute, Helmholtz Center for Polar and Marine Research, 27570 Bremerhaven, Germany

[5] University of Bremen, 28359 Bremen, Germany

***Corresponding authors E-mails:** mhallmaier@geomar.de; eva.rueckert@iup.uni-heidelberg.de

This PDF file includes: Supplementary text, Figures S1 to S11, SI References

Other supplementary materials for this manuscript: Dataset S1

1. Age models

For core PS 1768-8, we used the age model of Huang, et al. [1] for ages < 19 ka and > 116 ka. For the new measurements at core PS 1768-8, filling the gap of Huang, et al. [1] between 19 ka and 116 ka, the age models of [2,3] were refined by tuning of $\delta^{18}O$ from *N. pachyderma* [4] to δD from Vostok ice core on the AICC2012 chronology [5-7] (Fig. S1), in a similar manner as done by Huang et al. for Termination I and II. Therefore, we built a coherent dataset with the existing εNd measurements and age model optimizations of Huang and co-workers [1] verified by the matching εNd values at the tie points of both studies (see also fig. S4).

For ODP 1093 the age model was revised based on existing studies [8-10] and a graphical correlation of $\delta^{18}O$-data of *N. pachyderma* [11] to the Vostok ice core δD record on the AICC2012 chronology [5-7] (Fig. S2).

The tie points of the age models can be accessed in the attached Dataset S1. Due to the lack of foraminifera, $\delta^{18}O$ data is sparse at both sites and the graphical correlation can show uncertainties.

A few sediment samples (n = 19) of ODP 1093 are not from the shipboard splice. Using magnetic susceptibility and color reflectance data [11-13], we ensured that the meter-composite-depth (mcd) of these samples matches with the splice. However, minor differences could lead to deviations of up to ~3 ka (~0.5 m), as also discussed within the manuscript.

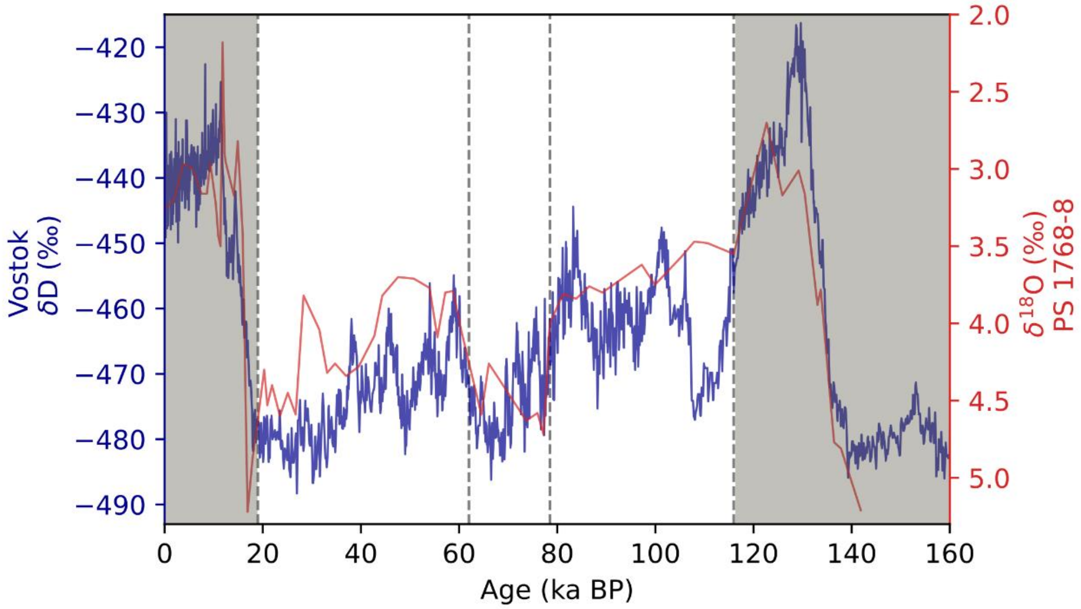

Fig. S1: Age model of core PS 1768-8. The blue curve shows the δD from Vostok ice core on the AICC2012 chronology [5-7], the red curve is the $\delta^{18}O$ from *N. pachyderma* of core PS 1768-8 [4]. Tie-Points marked by grey lines (see dataset 1). For Tie-Points younger than 19 ka and older than 116 ka (grey marked areas) see [1].

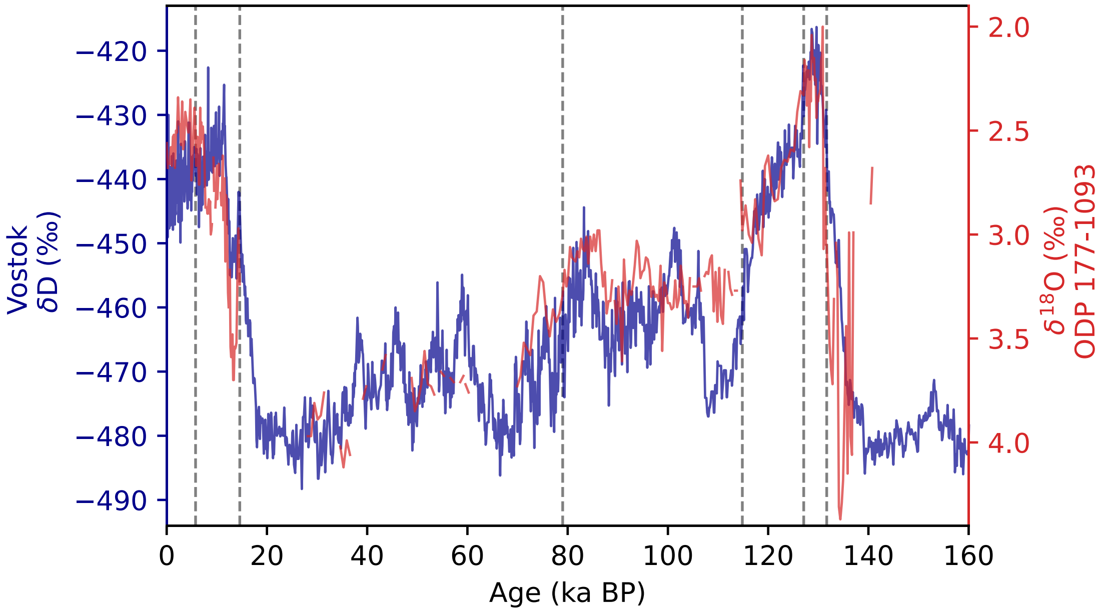

Fig. S2: Age model of core ODP 1093. The blue curve shows the δD from Vostok ice core on the AICC2012 chronology [5-7], the red curve is the $\delta^{18}O$-data of *N. pachyderma* of core ODP 1093 [11]. Tie-Points marked by grey lines (see dataset 1).

2. Test of the Leaching Protocol

The applied leaching protocol was compared to a later published very short 10s leaching protocol, to access the potential influence at the studied sites.

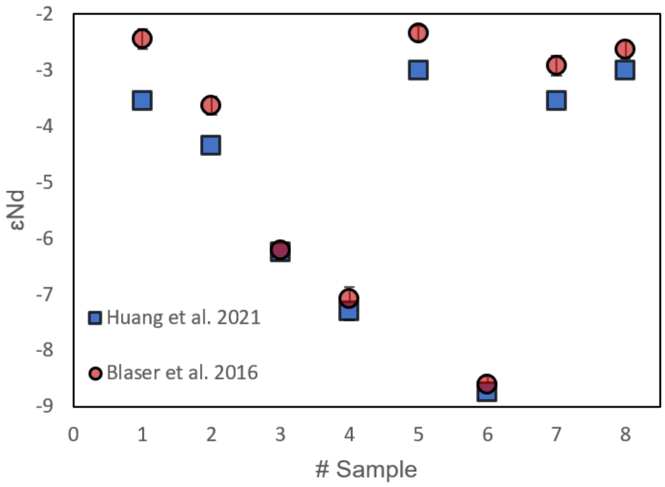

Fig. S3: Comparison of the used leaching protocol [14] and the 10s leaching protocol [15]. Small differences are observed for radiogenic samples. The maximal observed shift of 1.1 εNd is indicated as additional error bar in Fig. 2. This offset cannot explain the observed glacial-interglacial variability of up to 6 εNd.

3. Comparison between leachate and seawater Nd isotope compositions

The authigenic εNd records are compared with published nearby seawater values, planktonic foraminifera in a nearby core, picked opal values, detrital measurements and volcanic material.

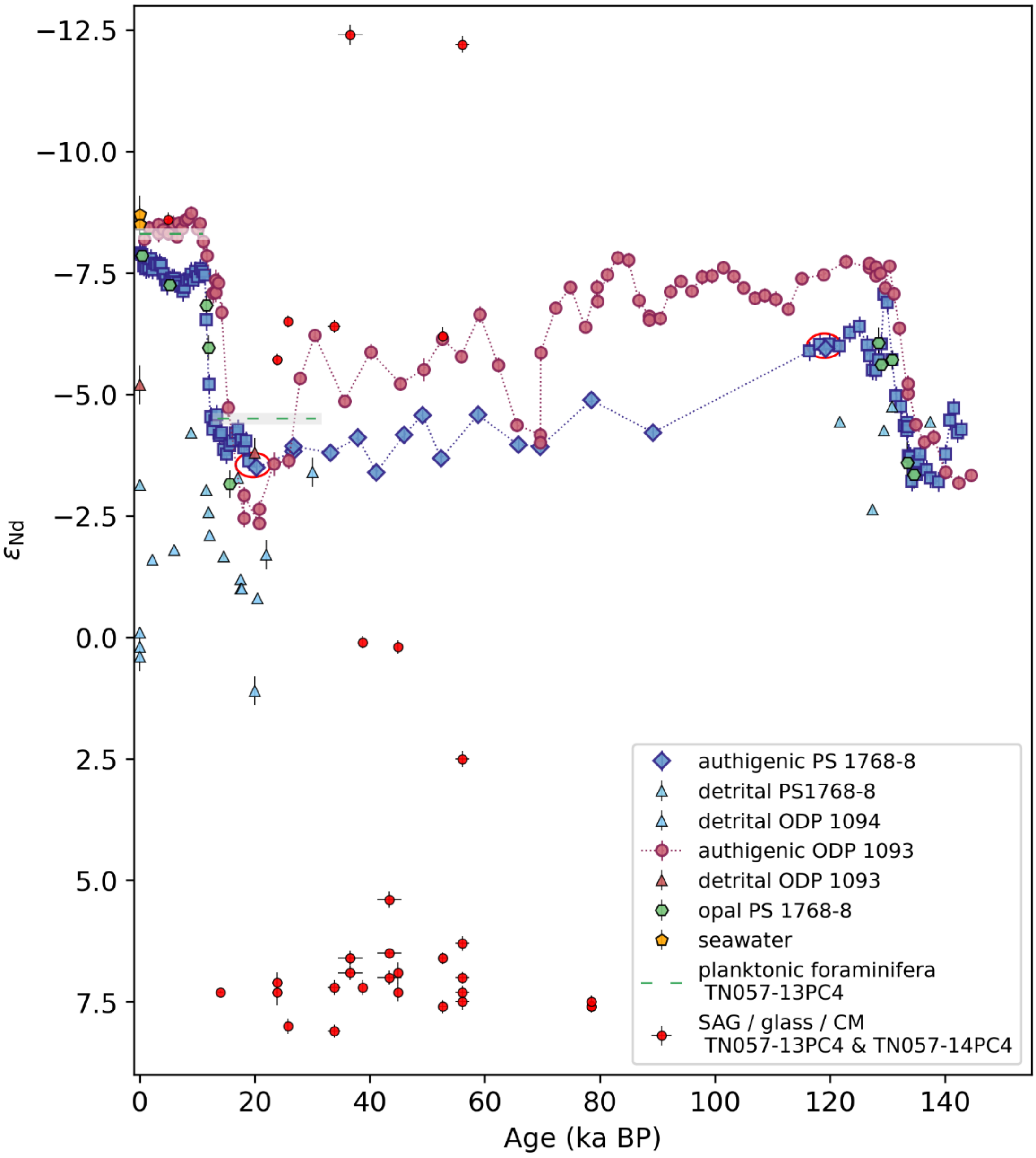

Fig. S4: The presented authigenic εNd records compared with published εNd data of different components. Comparison with nearby seawater data (station 104, 4440 m and station 113, 2400 m) [16], opal data of PS 1768-8 [1], compositions of Holocene and glacial-aged planktic foraminifera εNd measurements of nearby core TN057-13PC4 [17] shows that the leachates represent the authigenic fraction. Comparison with detrital εNd of ODP 1093[18], PS1768-8 [1], nearby site ODP 1094 [18,19] and volcanic material (single ash grains – SAG, glass, mixed clear minerals – CM) [17] shows that the observed trends in the authigenic εNd records of this study are not reflecting the detrital material or individual terrigenous components. Further, the red circles mark overlapping samples of authigenic εNd in PS1768-8 and demonstrate that the leached samples of this study (diamonds) are perfectly in line with another study/laboratory by Huang, et al. [1] (squares).

4. Assessing the effect of sediment composition

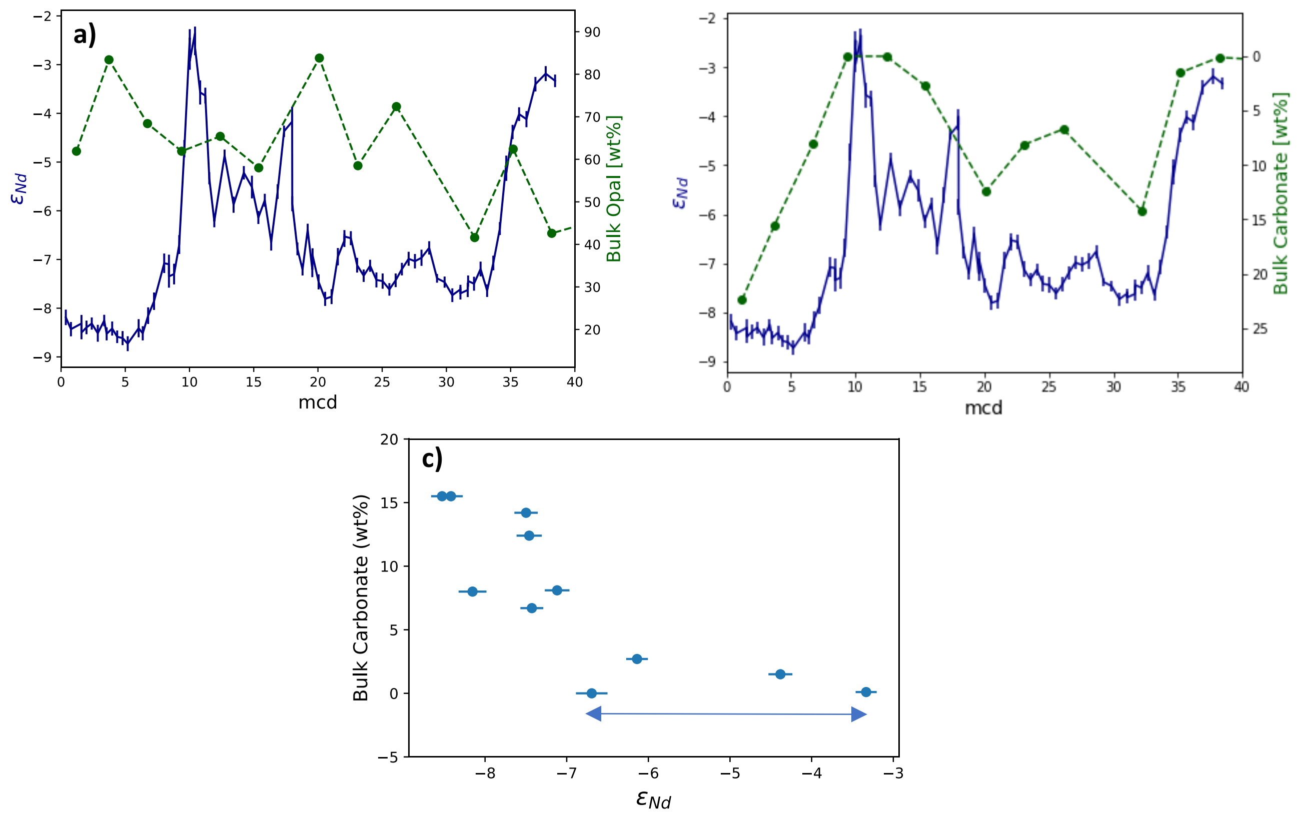

Fig S5: εNd at site ODP 1093 compared with bulk sediment composition (data from the scientific report of ODP Leg 177[13]) **a)** Bulk opal content **b, c)** Bulk carbonate content.

The sediment is characterized by high opal concentration and low carbonate content. The carbonate concentration is higher during interglacial periods. However, the εNd is not controlled by the carbonate content, as we observe almost the full observed range in εNd from -7 to -2 for carbonate free samples.

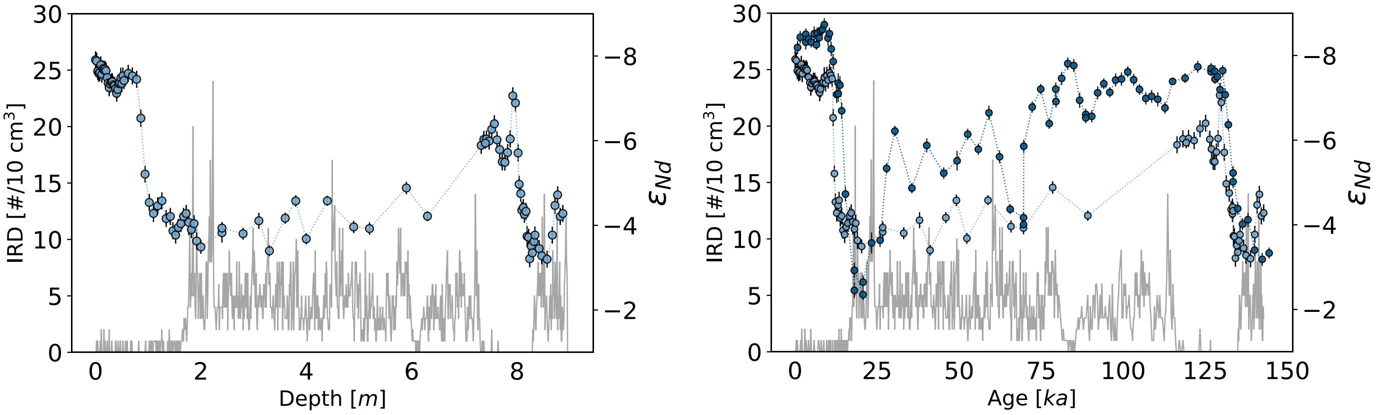

Fig S6: εNd compared with IRD content of PS1768-8[20].

The IRD input of the investigated sites is also climatically controlled. Glacial and cold periods are marked by higher IRD input at the sites compared to the interglacials. However, the sharp terminations observed in the Nd isotopic composition are not exactly coherent with the start of IRD input. We further measure the full range in εNd for sections with no IRD. Thus, the observed main climate signal is not controlled by IRD input. Nevertheless, the most radiogenic values at 20 ka BP might be to some extend influenced by a significant IRD event in this region.

5. Potential dust influence on Nd leaching

Dust input (as indicated by Fe fluxes) is increased during colder climates[8,21], as it is also controlled by glacial-interglacial changes. Thus, the εNd signature and the dust input are climatically controlled and a strong correlation is not surprising, as seen at ODP Site 1093.

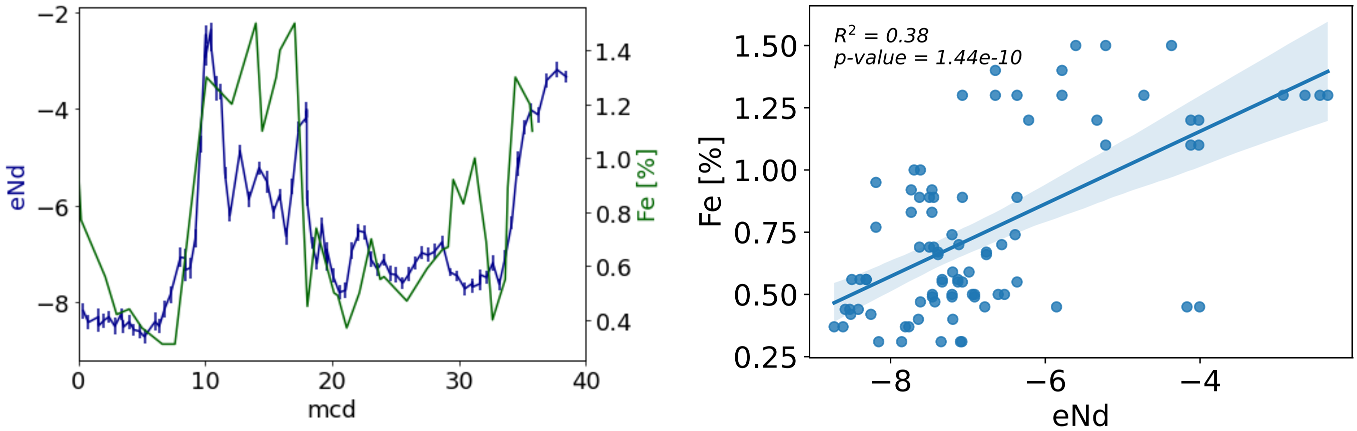

Fig S7: **a)** εNd and Fe concentration as a measure of dust concentration in the sediment of ODP Site 1093. Fe data from [8]. **b)** Linear regression of iron concentration vs. εNd.

However, we can clearly identify two groups, one with high dust input (mainly glacials) and one with low dust input (mainly interglacial). If we perform the linear regression on the two groups separately, the correlation vanishes, which indicates a case of the Simpson's paradox.

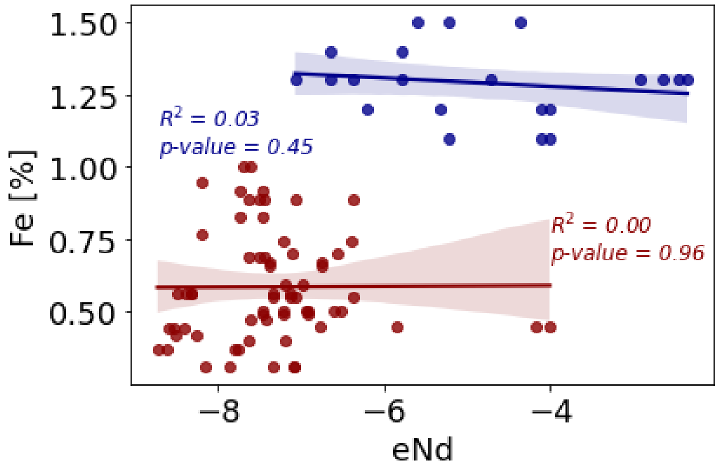

Fig S8: Linear regression for separate clusters of high and low Fe content.

Thus, the measured leaches are independent from changes in the dust content or sediment composition. Almost the whole range in εNd was measured regardless of whether the sediment section bears high or low Fe content. An observed correlation is therefore not necessarily a causal consequence or explanation; both signals are rather simply climatically controlled.

6. Model experiment

To further test the hypothesis of a large-scale Pacific intrusion into the Atlantic Section of the Southern Ocean, we run an Ocean general circulation model simulation (FESOM2.0). A tracer was released in the Equatorial East Pacific (seafloor to 1000m), and compared for a present day and LGM run.

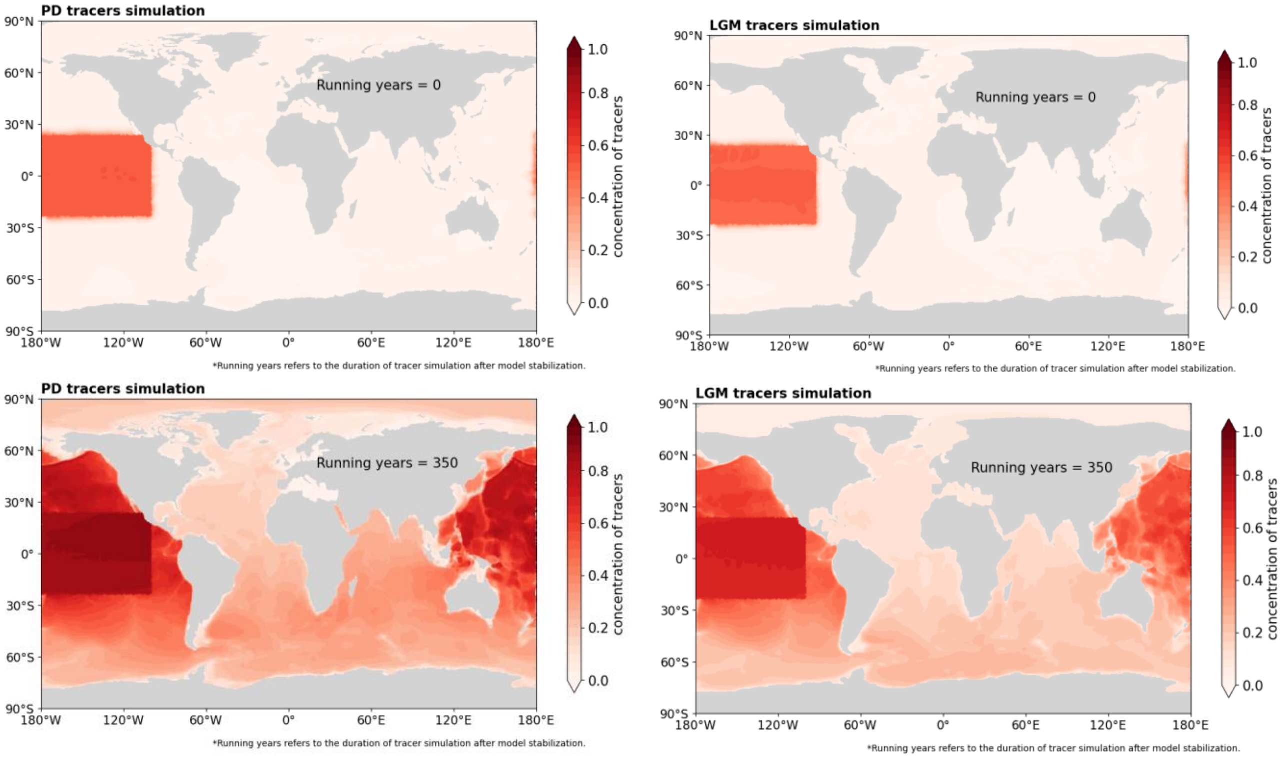

Fig. S9: Initial tracer distribution and after 350 running years after model stabilization.

7. εNd and authigenic Pb across Termination I

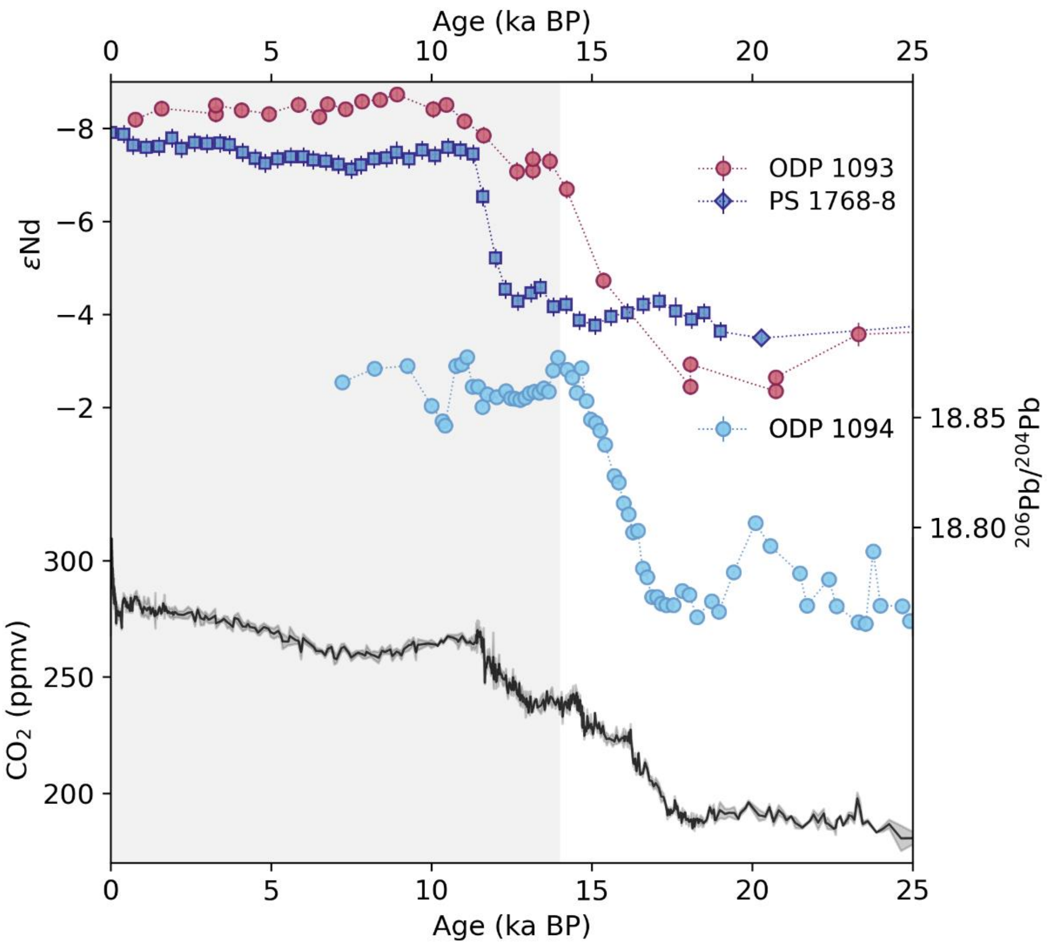

Fig. S10: εNd at Site ODP 1093 and PS1768-8 [1] and authigenic Pb at Site ODP 1094[1] across Termination I. Shift in εNd at ODP 1093 starts earlier than in PS1768-8 and in two steps. The first step is synchronous to the shift in authigenic Pb (ODP 1094) and the second one synchronous to the change in εNd in PS1768-8.

8. εNd data compilation

To obtain more information about the water mass distribution in different depths and to place our results in a larger perspective, existing εNd data of the SO and South Atlantic south of 29°S were compiled and extended by the novel results of this study to develop glacial and interglacial εNd depth profiles of the Atlantic section of the SO (Fig. 6, S10). The core locations, water depths, εNd data and references can be found in Dataset S1. The influences of various locations must be considered, as latitudinal and longitudinal differences lead to variations in the εNd signal. For example, core locations further north in the Cape Basin are reached from a much higher amount of NADW flowing along the eastern Atlantic margin southward. This leads to less radiogenic εNd values. The data was limited to locations east of the Drake-Passage and west of Cape Agulhas (20°E), as well as latitudes south of 29°S. All these locations are within a similar Coriolis driven flow path of water moving north and south.

To complete the lack of sediment derived εNd data from < 1000 m water depth, we include cold-water coral εNd data for the upper water column from the Drake Passage [22].

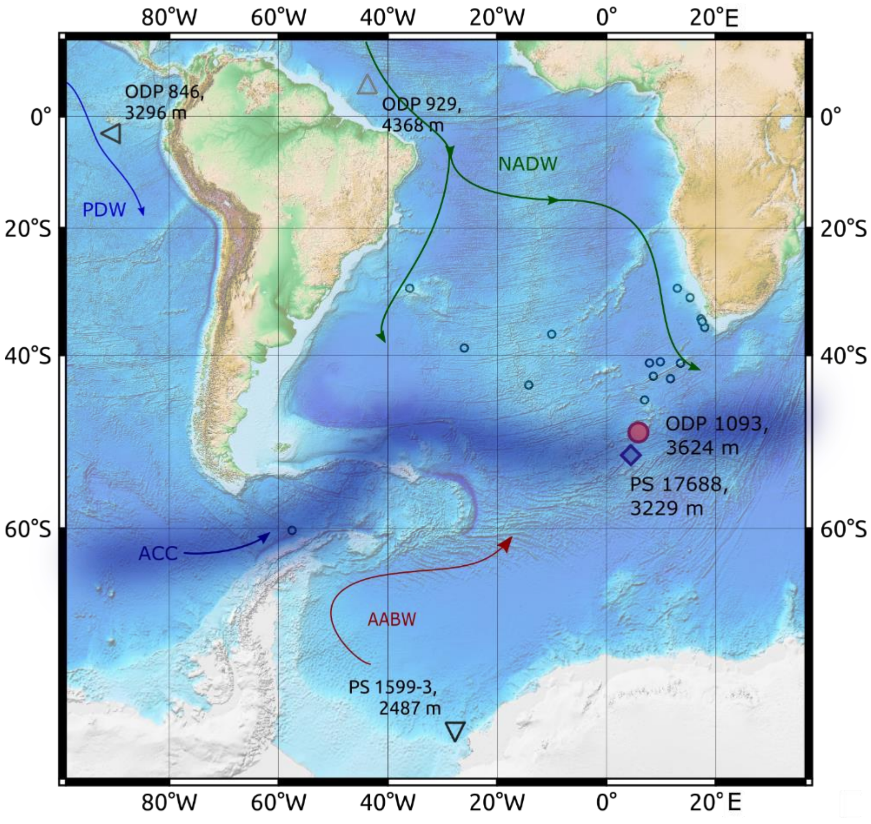

Fig. S11: Overview map of the core sites, which are shown with similar symbols in Figure 2. The small circles show the different core sites used for the compilation leading to Figure 6. Arrows show the main deep water pathways. The bathymetry is based on ETOPO1 Global Relief Model [23].

Dataset S1 (separate file): Excel file containing measured εNd data, core compilation and age model tie points.

References:

1 Huang, H., Gutjahr, M., Eisenhauer, A. & Kuhn, G. No detectable Weddell Sea Antarctic Bottom Water export during the Last and Penultimate Glacial Maximum. *Nat Commun* **11**, 424, doi:10.1038/s41467-020-14302-3 (2020).

2 Frank, M., Mangini, A., Gersonde, R., van der Loeff, M. R. & Kuhn, G. Late Quaternary sediment dating and quantification of lateral sediment redistribution applying $^{230}Th_{ex}$: A study from the eastern Atlantic sector of the Southern Ocean. *Geologische Rundschau* **85**, 554-566 (1996).

3 Frank, M. *et al.* Similar glacial and interglacial export bioproductivity in the Atlantic Sector of the Southern Ocean: Multiproxy evidence and implications for glacial atmospheric CO2. *Paleoceanography* **15**, 642-658, doi:10.1029/2000pa000497 (2000).

4 Niebler, H.-S. in *In supplement to: Niebler, H-S (1995): Rekonstruktionen von Paläo-Umweltparametern anhand von stabilen Isotopen und Faunen-Vergesellschaftungen planktischer Foraminiferen im Südatlantik = Reconstruction of paleo-environmental parameters using stable isotopes and faunal assemblages of planktonic foraminifera in the South Atlantic Ocean. Berichte zur Polarforschung = Reports on Polar Research, 167, 198 pp,* https://doi.org/10.2312/BzP_0167_1995 (PANGAEA, 2002).

5 Petit, J. R. *et al.* Climate and atmospheric history of the past 420,000 years from the Vostok ice core, Antarctica. *Nature* **399**, 429-436, doi:10.1038/20859 (1999).

6 Bazin, L. *et al.* An optimized multi-proxy, multi-site Antarctic ice and gas orbital chronology (AICC2012): 120-800 ka. *Clim. Past* **9**, 1715-1731, doi:10.5194/cp-9-1715-2013 (2013).

7 Veres, D. *et al.* The Antarctic ice core chronology (AICC2012): an optimized multi-parameter and multi-site dating approach for the last 120 thousand years. *Clim. Past* **9**, 1733-1748, doi:10.5194/cp-9-1733-2013 (2013).

8 Lal, D. *et al.* Paleo-ocean chemistry records in marine opal: Implications for fluxes of trace elements, cosmogenic nuclides (10Be and 26Al), and biological productivity. *Geochimica et Cosmochimica Acta* **70**, 3275-3289, doi:10.1016/j.gca.2006.04.004 (2006).

9 Schneider-Mor, A. *et al.* Nutrient regime at the siliceous belt of the Atlantic sector of the Southern Ocean during the past 660 ka. *Paleoceanography* **23**, doi:10.1029/2007pa001466 (2008).

10 Schneider Mor, A. *et al.* Variable sequence of events during the past seven terminations in two deep-sea cores from the Southern Ocean. *Quaternary Research* **77**, 317-325, doi:10.1016/j.yqres.2011.11.006 (2012).

11 Hodell, D. A., Charles, C. D., Curtis, J. H., Mortyn, P. G., Ninnemann, U. S., and Venz, K. A. in *Proceedings of the Ocean Drilling Program, 177 Scientific Results* Vol. 177 (ed R. Gersonde, Hodell, D. A., and Blum, P) (2003).

12 Gersonde, R., Hodell, D.A., Blum, P. Proc. ODP, Init. Repts., 177. *College Station, TX (Ocean Drilling Program)*, doi:10.2973/odp.proc.ir.177.1999 (1999).

13 Gersonde, R., Hodell, D.A., and Blum, P. (Eds.). Proc. ODP, Sci. Results, 177. *College Station, TX (Ocean Drilling Program)*, doi:doi:10.2973/odp.proc.sr.177.2003 (2003).

14 Blaser, P. *et al.* Extracting foraminiferal seawater Nd isotope signatures from bulk deep sea sediment by chemical leaching. *Chemical Geology* **439**, 189-204, doi:10.1016/j.chemgeo.2016.06.024 (2016).

15 Huang, H., Gutjahr, M., Kuhn, G., Hathorne, E. C. & Eisenhauer, A. Efficient Extraction of Past Seawater Pb and Nd Isotope Signatures From Southern Ocean Sediments. *Geochemistry, Geophysics, Geosystems* **22**, doi:10.1029/2020gc009287 (2021).

16 Stichel, T., Frank, M., Rickli, J. & Haley, B. A. The hafnium and neodymium isotope composition of seawater in the Atlantic sector of the Southern Ocean. *Earth and Planetary Science Letters* **317-318**, 282-294, doi:10.1016/j.epsl.2011.11.025 (2012).

17 Nielsen, S. H. H., Hodell, D. A., Kamenov, G., Guilderson, T. & Perfit, M. R. Origin and significance of ice-rafted detritus in the Atlantic sector of the Southern Ocean. *Geochemistry, Geophysics, Geosystems* **8**, doi:10.1029/2007GC001618 (2007).

18 Noble, T. L. *et al.* Greater supply of Patagonian-sourced detritus and transport by the ACC to the Atlantic sector of the Southern Ocean during the last glacial period. *Earth and Planetary Science Letters* **317-318**, 374-385, doi:10.1016/j.epsl.2011.10.007 (2012).

19 Latimer, J. C., Filippelli, G. M., Hendy, I. L., Gleason, J. D. & Blum, J. D. Glacial-interglacial terrigenous provenance in the southeastern Atlantic Ocean: The importance of deep-water sources and surface currents. *Geology* **34**, doi:10.1130/g22252.1 (2006).

20 Diekmann, B. *et al.* in *The South Atlantic in the Late Quaternary* 375-399 (Springer, 2003).

21 Martínez-García, A. *et al.* Iron Fertilization of the Subantarctic Ocean During the Last Ice Age. *Science* **343**, 1347-1350, doi:doi:10.1126/science.1246848 (2014).

22 Wilson, D. J. *et al.* Sea-ice control on deglacial lower cell circulation changes recorded by Drake Passage deep-sea corals. *Earth and Planetary Science Letters* **544**, 116405, doi:https://doi.org/10.1016/j.epsl.2020.116405 (2020).

23 Amante, C. & Eakins, B. ETOPO1 1 Arc-Minute Global Relief Model: Procedures, Data Sources and Analysis, National Geophysical Data Center, NESDIS, NOAA, US Dept. *Commerce, Boulder, CO, USA*, doi:doi:10.7289/V5C8276M (2009).
